# *In vitro* neutralization of IL-6 receptor exacerbates damage to intestinal epithelial cells during *Mycobacterium avium paratuberculosis* infection

**DOI:** 10.3389/fimmu.2024.1412800

**Published:** 2024-08-07

**Authors:** Ala’ Alhendi, Saleh A. Naser

**Affiliations:** Division of Molecular Microbiology, Burnett School of Biomedical Sciences, College of Medicine. University of Central Florida, Orlando, FL, United States

**Keywords:** IL-6, MAP, Crohn’s disease, intestinal epithelial injury, paratuberculosis, IBD

## Abstract

Like TNFα, IL-6 is upregulated in Crohn’s disease (CD) especially in patients associated with *Mycobacterium avium paratuberculosis* (MAP) infection, and both cytokines have been targeted as a therapeutic option for the treatment of the disease despite the accepted partial response in some patients. Limited response to anti-IL-6 receptor-neutralizing antibodies therapy may be related to the homeostatic dual role of IL-6. In this study, we investigated the effects and the signaling mechanism of IL-6 involved in intestinal epithelial integrity and function during MAP infection using an *in vitro* model that consists of THP-1, HT-29 and Caco-2 cell lines. Clinically, we determined that plasma samples from MAP-infected CD patients have higher IL-6 levels compared to controls (P-value < 0.001). In CD-like macrophages, MAP infection has significantly upregulated the secretion of IL-6 and the shedding of (IL-6R) from THP-1 macrophages, P-value < 0.05. Intestinal cell lines (Caco-2 and HT-29) were treated with the supernatant of MAP-infected THP-1 macrophages with or without a neutralizing anti-IL-6R antibody. Treating intestinal Caco-2 cells with supernatant of MAP-infected macrophages resulted in significant upregulation of intestinal damage markers including claudin-2 and SERPINE1/PAI-1. Interestingly, blocking IL-6 signaling exacerbated that damage and further increased the levels of the damage markers. In HT-29 cells, MAP infection upregulated MUC2 expression, a protective response that was reversed when IL-6R was neutralized. More importantly, blocking IL-6 signaling during MAP infection rescued damaged Caco-2 cells from MAP-induced apoptosis. The data clearly supports a protective role of IL-6 in intestinal epithelia integrity and function especially in CD patients associated with MAP infection. The findings may explain the ineffective response to anti-IL6 based therapy and strongly support a therapeutic option that restores the physiologic level of IL-6 in patient’s plasma. A new treatment strategy based on attenuation of IL-6 expression and secretion in inflammatory diseases should be considered.

## Introduction

Crohn’s disease (CD) is a chronic and progressive inflammatory bowel disease (IBD) that affects any part of the gastrointestinal tract (1). The incidence and prevalence of CD globally has been rising in the past several decades with an estimated 1.011 million CD patients in USA as reported in 2023 (2, 3). This growing number of cases imposes an expanding burden on the economy and healthcare systems to accommodate this costly and chronic debilitating disorder (3, 4). Clinically, CD presents with abdominal pain, bowel obstruction or diarrhea, fever, weight loss and fatigue (1, 5). The etiology of CD is complex with environmental triggers, genetic predisposition and immunological factors contributing to the disease onset and progression (6). This multifactorial nature of the disease leads to disruption of the gut homeostasis evidenced by increased intestinal permeability, imbalanced microbiome (dysbiosis) and unusual inflammatory responses. The disruption of the intestinal barrier facilitates luminal bacterial translocation and the consequent perpetuation of inflammation (7). *Mycobacterium avium paratuberculosis* (MAP) is a leading infectious agent candidate to cause this chronic inflammation in CD patients and is extensively studied in our lab to model the disease *in vitro*. MAP has been isolated and cultured from the tissues of CD patients at higher rates compared to controls (8).

Among many inflammatory cytokines upregulated during CD intestinal inflammation, IL-6 is of great interest because of its dual nature in intestinal homeostasis and pathology (9). IL-6 is essential for the integrity of the intestinal epithelial tissue. It is important to regulate mucin production by goblet cells and to maintain the barrier function and controlled permeability of the intestinal layer. Absence of IL-6 is detrimental to the protective intestinal mucous layer and to the intricately regulated tight junctions which regulate intestinal paracellular transport (10). Moreover, IL-6 is important for intestinal tissue repair as it sustains the stem cell niche and facilitates its proliferation to replace cells after intestinal injury (11, 12). During intestinal inflammation, the upregulated levels of IL-6 lead to epithelial damage and sustenance of the chronic inflammation. IL-6 compromises the permeability of the intestinal epithelial barrier making it leaky to cations, a feature of exudative diarrhea seen in IBD patients (13–15). Moreover, IL-6 can function via the trans-signaling pathway where soluble IL-6 receptor (sIL-6R) activates gp130-expressing target cells after being activated by IL-6 binding. This leads to prolonging the chronic inflammation by rendering T-cells resistance to apoptosis (16).

IL-6 has been targeted for the treatment of CD and other inflammatory conditions. Although CD patients demonstrated a good response in terms of disease activity index and remission, there was no improvement in the intestinal histological appearance as an important treatment outcome for a sustained remission (17, 18). Rheumatoid arthritis patients treated with an anti-IL-6R antibody (tocilizumab), and CD patients treated with anti-IL-6 antibody (PF-04236921) experienced gastrointestinal perforations and bleeding which was the reason behind terminating these trials (19, 20).

In this study, we demonstrate the detrimental effects of blocking IL-6 signaling during MAP infection on intestinal epithelial integrity and function. We present this as we compare to the direct damaging effects of recombinant IL-6 (rIL-6) treatment on cultured intestinal cells. The data presented here should help understanding the adverse effects of IL-6-targeted therapies in CD patients and highlights the need for an alternative therapy that restores the physiological level of IL-6 in patients associated with MAP infection.

## Materials and methods

### Clinical samples

Peripheral blood samples were collected from 32 CD patients and 14 healthy individuals (4.0 mL K2-EDTA tubes). The status of MAP infection in these samples was assessed using IS900 nested PCR (nPCR) as described earlier (21). Plasma separated from blood samples was analyzed to determine the concentration of IL-6 using the Human IL-6 (2^nd^ gen) Assay cartridge (Bio-Techne, Minneapolis, MN) by the Ella automated ELISA system (Bio-Techne, Minneapolis, MN). This study was approved by the University of Central Florida Institutional Review Board #STUDY00003468.

### Cell lines and culture conditions

THP-1 monocyte (ATCC TIB-202) were cultured in RPMI-1640 (ATCC, 30-2001) supplemented with 0.05 mM 2-mercaptoethanol and 10% fetal bovine serum (FBS; Sigma Life Science, St. Louis, MO). For experiments, cells were seeded at 5 X 10^5^ cells/mL in a 12-well plate and differentiated to macrophages by adding 50 ng/mL phorbol 12-myristate 13-acetate (PMA; Sigma Life Science, St. Louis, MO) and incubated at 37°C in the presence of 5% CO_2_ for 48 hours. Primed THP-1 macrophages were either left as controls or infected with 1x10^7^ CFU/mL of MAP strain UCF4 for 24 hours before RNA extraction and supernatant collection. For intestinal cells, we used the human enterocytic cell line, Caco-2 (ATCC HTB-37) and the mucin-producing HT-29 (ATCC HTB-28) cell line. Caco-2 cells were cultured in Eagle’s minimum essential medium (ATCC, 30-2003) supplemented with 20% FBS. For experiments, cells were seeded at 3.5 X 10^5^ cells/mL in 12-well plates and allowed to differentiate for 2-3 weeks with media being changed every other day. HT-29 were maintained in ATCC-formulated McCoy’s 5a medium modified (30-2007) supplemented with 10% FBS. They were seeded at 3.5 X 10^5^ cells/mL in 12-well plates and allowed to differentiate for 3 weeks with media being changed every other day. All cells were kept in a humidified incubator at 37°C and in the presence of 5% CO_2_. Intestinal cells were treated with different concentrations of recombinant IL-6 (Bio-Techne, Minneapolis, MN) or recombinant TNFα (MilliporeSigma, Burlington, MA) or a combination of both at 5 ng/mL. Cell lines without any treatment or infection were always included as controls.

### Measurement of IL-6 and sIL-6R in MAP-infected THP-1

The supernatants of THP-1 control cells and MAP-infected groups were collected after 24 and 48 hours of infection. ELISA assays for IL-6 and sIL-6R (Thermo Fisher Scientific, Waltham, MA) were performed per manufacturer’s instructions. Results were read at an absorbance of 450 nm using a Multiskan FC plate reader (Thermo Fisher Scientific, Waltham, MA). All treatment groups were tested in 4 technical repeats.

### Treatment with IL-6 receptor-neutralizing antibodies

Differentiated THP-1 macrophages were infected with 1x10^7^ CFU/mL of MAP strain UCF4 for 24 hours. The supernatant of these infected macrophages was collected and used for treatment of Caco-2 and HT-29 intestinal cell lines. Intestinal cells were treated with anti-IL6R monoclonal antibodies at 0.5 or 1 µg/mL final concentration (Bio-Techne, Minneapolis, MN) for 1 hour at cell culture conditions (37°C and 5% CO_2_). Simultaneously, 0.5 or 1 µg/mL of anti-IL-6R were added to 2 mL of MAP-infected THP-1 supernatant and incubated at room temperature (22°C) on a shaker for 1 hour. MAP-infected THP-1 supernatants blocked with anti-IL-6R were then used to replace the media on the corresponding intestinal epithelial cells that have also been treated with the same concentration of anti-IL-6R (double blocking). Intestinal cells and their supernatants were then harvested for RNA extraction and qRT-PCR or for other epithelial integrity assays.

### RNA extraction, reverse transcription, and qRT-PCR

Cellular RNA was extracted from control and treated cells using RNeasy^®^ Mini Kit (Qiagen, Hilden, Germany) following manufacturer’s instructions. RNA (1000 ng) was used in a reverse transcription reaction to generate cDNA using the high-capacity cDNA reverse transcription kit (Thermo Fisher Scientific, Waltham, MA). cDNA was diluted 1:25 in nuclease-free water and 5 µL were added to 15 µL of a reaction mixture made up of: 10 µL of PowerUp™ SYBR™ Green MasterMix (Thermo Fisher Scientific, Waltham, MA), 1 µL of the forward and reverse primers; *SERPINE1*, *NOX-1*, *CLDN2* and *MUC2* (Bio-Rad, Hercules, CA), and lastly 4 µL of nuclease-free water (Life Technologies, Carlsbad, CA) in 96-well MicroAmp^®^ PCR plates (Life Technologies, Carlsbad, CA). We used GAPDH in all PCR runs as the housekeeping gene for baseline cycle threshold (Ct) readings. The real-time PCR reaction was carried out in a 7500 Fast Real-Time PCR system (Applied Biosystems, Waltham, MA). The relative mRNA expression of each gene was expressed as a fold change via the equation: 2^(-ΔΔCt). Each PCR reaction was performed in technical triplicates.

### Measurement of apoptosis in Caco-2 using annexin V-based luminescence assay

Caco-2 were seeded at 200µL of 3.5 X 10^5^ cells/mL in 96-well opaque-sided plates. After 14-21 days of differentiation, cells were treated with rIL-6 (0.16 - 10 ng/mL) or with MAP-infected THP-1 supernatant double blocked with anti-IL-6R as described earlier for 24 hours. Control wells were left untreated. We used the annexin V-based luminescence assay from Promega™, RealTime-Glo™ Apoptosis Assay (Madison, WI) to assess apoptosis levels following manufacturer’s instructions. Luminescence was recorded using the Promega™ GloMax Navigator system GM-2000 (Madison, WI).

### Dihydroethidium staining for oxidative stress visualization

HT-29 cells were cultured in a Falcon™ Chambered Cell Culture Slide (Thermo Fisher Scientific, Waltham, MA) for the regular period of differentiation. After 24 hours of treatment with rIL-6 or anti-IL-6R double-blocked MAP infection, cells were washed with PBS and fixed on the slides with 10% formalin. Next, they were treated with 1µM DHE stain (Thermo Fisher Scientific, Waltham, MA) for 25 minutes. After that, 4′,6-diamidino-2-phenylindole (DAPI; Vector Laboratories, Burlingame, CA) was used to stain nuclei. Slides were examined under an Amscope IN480TC-FL-MF603 fluorescent microscope as described previously in (22). NIH imageJ program was used to merge DAPI and DHE stain images.

### Measurement of PAI-1 released by Caco-2

Supernatants were collected from Caco-2 cells treated with MAP-infected THP-1 supernatants whether they were left unblocked or treated with different concentrations of anti-IL-6R. PAI-1 levels were measured in these supernatants using the PAI-1 ELISA Assay (ThermoFisher, Waltham, MA) following manufacturer’s instructions. Absorbance was read at a wavelength of 450 nm using a Multiskan FC plate reader (Thermo Fisher Scientific, Waltham, MA). All treatment groups were tested in 4 technical repeats.

### Measurement of transepithelial electrical resistance

Caco-2 cells were grown on 12-well plate inserts with a pore size of 0.3 µm at 1 mL of 3.5 X 10^5^ cells/mL (Greiner Bio-One, Kremsmünster, Austria). After differentiation, cells were treated with their respective treatment. TEER measurements were taken at baseline and 24 hours after treatment using the Millicell^®^ ERS-2 electrical resistance system (MilliporeSigma, Burlington, MA) as described earlier (13). Resistance values are presented as % of the baseline value.

### Statistical analysis

For statistical significance analysis, we used the two-tailed nonparametric sample t-test or the two-way analysis of variance (ANOVA). All analysis was carried out on the GraphPad Prism V.7.02 software (GraphPad, La Jolla, CA). All experiments were performed in biological triplicates or quadruplicates. Results are expressed as mean ± SD. *Indicates P-values less than 0.05, **indicates P-values less than 0.005 and ***indicates P-values less than 0.001.

## Results

### IL-6 is elevated in CD plasma

**Figure 1:**
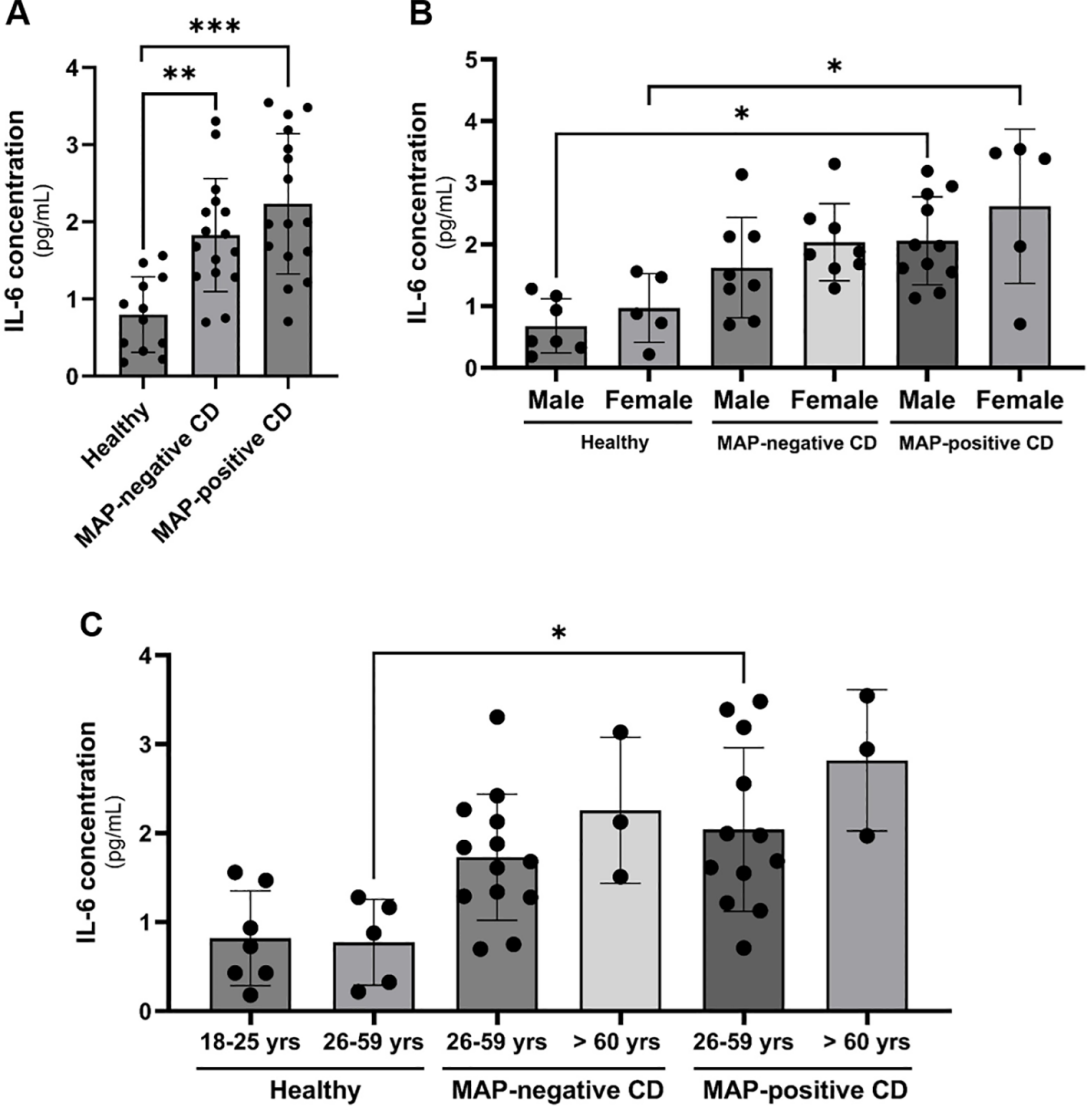
Clinical IL-6 levels in CD patients’ plasma with or without a MAP infection compared to healthy control subjects matched based on gender and age group. **(A–C)** IL-6 levels were measured using ELISA in plasma samples from healthy individuals (N=12), MAP-negative CD patients (N=16) and MAP-positive CD patients (N=16). *Indicates P-value < 0.05, **indicates P-value < 0.005 and *** indicates P-value < 0.001.

We assessed the levels of systemic IL-6 release in plasma samples from 12 healthy individuals, 16 MAP-negative CD patients and 16 MAP-positive CD patients. The plasma samples included in this study were tested for the presence of MAP DNA by nested PCR as described in our earlier study (21). We included plasma samples from CD patients that were tested positive or negative for MAP and plasma samples from healthy control that tested negatively for MAP. As shown in **Figure 1A**, a doubling of circulating IL-6 levels especially in the plasma of MAP-positive CD group is seen compared to healthy individuals. To account for variations in cytokine profiles based on age and gender, we stratified the results based on gender and age group (18-25 years old, 26-59 years old and > 60 years old) as shown in **Figures 1B** and **C**. Clustering patients based on gender shows a significant upregulation in plasma IL-6 levels in MAP-positive CD patients compared to healthy individuals (Males: 2.06 ± 0.71 pg/mL vs. 0.68 ± 0.44 pg/mL, females: 2.62 ± 1.25 pg/mL vs. 0.97 ± 0.55 pg/mL, respectively), P-value < 0.05. There was no statistical significance when gender-matched MAP-negative groups were compared to healthy individuals. Moreover, we classified study subjects based on their age group: 18-25 year-olds, 26-59 year-olds and > 60 year-olds. This resulted in partial inconsistency between the different study groups as the young group (18-25 year-olds) was absent from CD patients and the old group (> 60 year-olds) was absent from the healthy cohort. However, when comparing the same age groups, MAP-positive CD patients in the 26-59 years group have a significant increase in circulatory IL-6 compared to healthy individuals of the same age group (2.04 ± 0.92 pg/mL vs 0.77 ± 0.48 pg/mL, P-value < 0.05). There was no statistical significance when age-matched MAP-negative groups were compared to healthy individuals in the 26-59 year group.

### IL-6 and sIL-6R are elevated in MAP-infected macrophages (CD-like macrophages)

**Figure 2:**
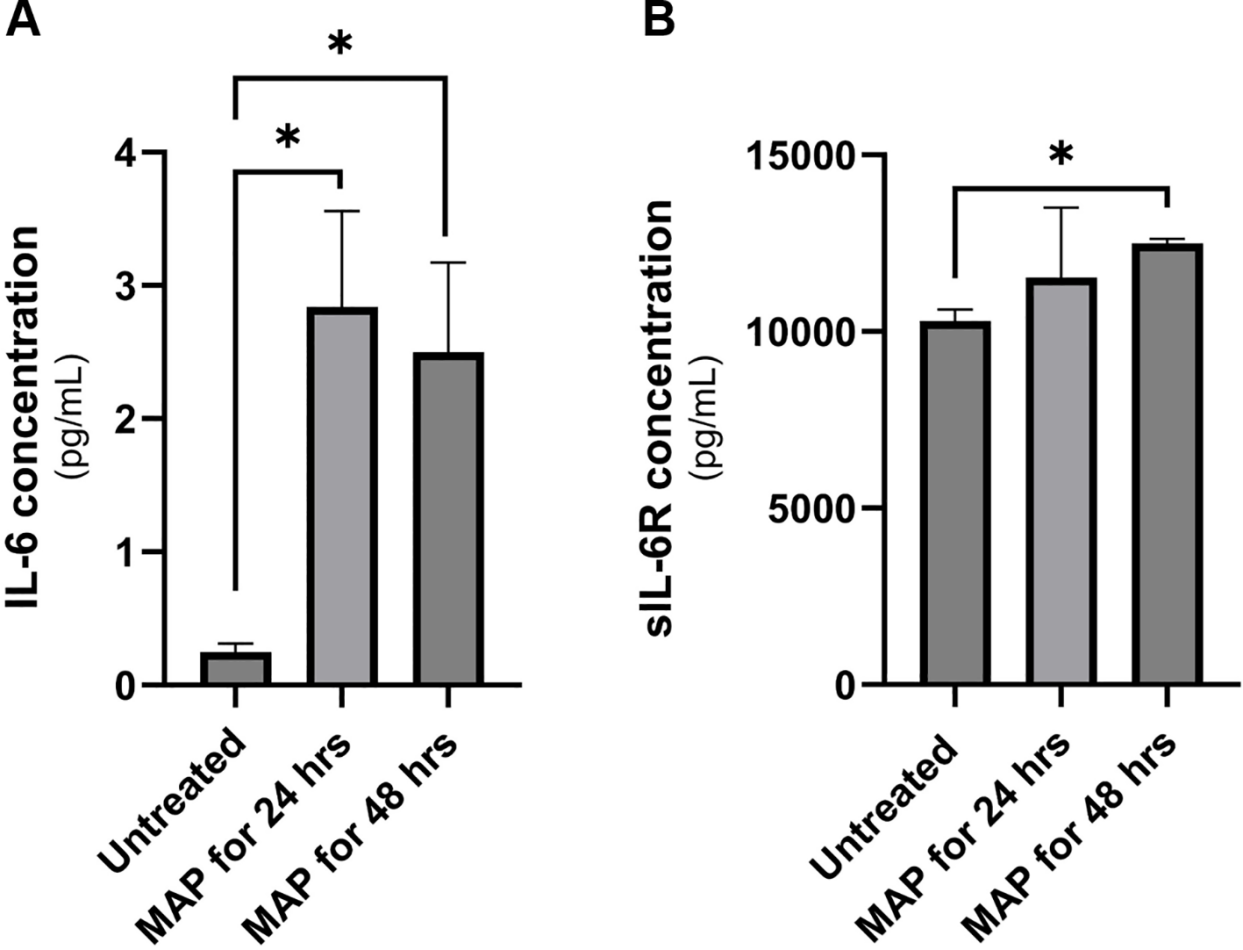
IL-6 and sIL-6R release levels in MAP-infected THP-1 macrophages. Supernatants of MAP-infected THP-1 macrophages were used in two separate ELISA assays for for IL-6 **(A)** and sIL-6R **(B)** measurement. *Indicates a P-value < 0.05.

Supernatant of THP-1 macrophages was collected following 24 or 48 hours of MAP infection and used in two separate ELISAs to determine the concentration of IL-6 and sIL-6R. As shown in **Figure 2A**, MAP infection induces the upregulation of IL-6 released by macrophages when measured after 24 hr and 48 hr of infection (2.8 ± 0.72 pg/mL and 2.5 ± 0.67 pg/mL, respectively) compared to untreated controls (0.25 ± 0.06 pg/mL). As shown in **Figure 2B**, the shedding of IL-6R in infected macrophages was measures at 11,525 ± 1986 pg/mL (24-hour infection), and 12,497 ± 122.7 pg/mL (48-hour infection) compared to 10,300 ± 321.3 pg/mL in uninfected macrophages (P-value < 0.05). Both findings indicate an upregulation in IL-6 signaling during MAP infection which will potentially have an influence on intestinal epithelia.

### Treatment of intestinal epithelial cells with rIL-6 modulates MUC2 and CLDN2 expression

**Figure 3:**
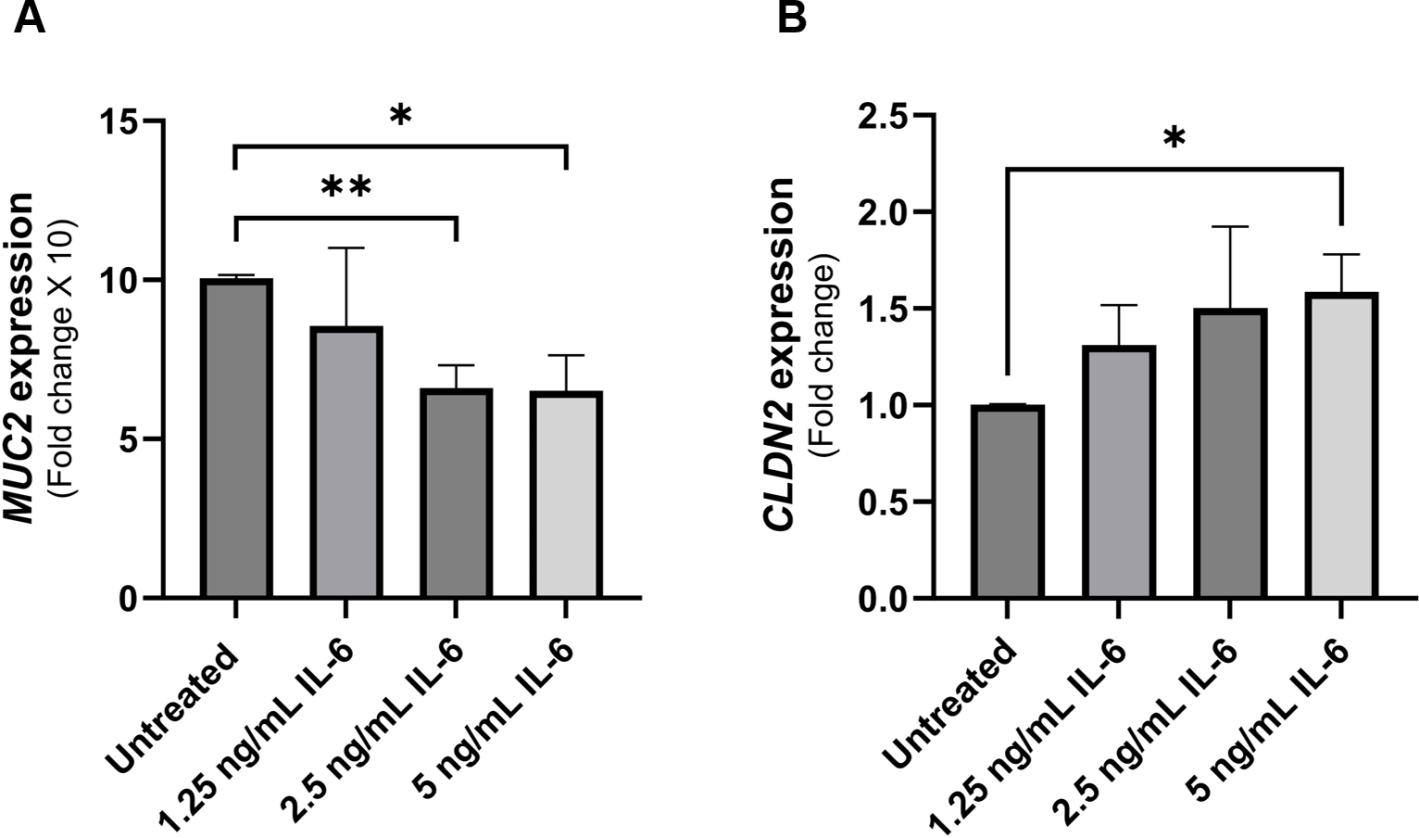
Effect of rIL-6 treatment on intestinal epithelial integrity markers. **(A)** HT-29 cells were treated with different concentrations of rIL-6 (1.25, 2.5 and 5 ng/mL) for 24 hours followed by qRT-PCR for MUC2 gene expression. **(B)** Caco-2 cells were treated with different concentrations of rIL-6 (1.25, 2.5 and 5 ng/mL) for 24 hours followed by qRT-PCR for CLDN2 gene expression. *Indicates P-value < 0.05 and **indicates P-value < 0.005.

Mucin 2 (MUC2) is the predominant gel-forming mucin in the intestines (23). In HT-29 mucin-producing cell line, we show a dose-dependent reduction in MUC2 gene expression (reduction by 34% at 2.5 ng/mL rIL-6, P-value < 0.005, and reduction by 35% at 5 ng/mL rIL-6, P-value < 0.05) as shown in **Figure 3A**. On the other hand, rIL-6 enhanced the expression of the leaky epithelial tight junction protein known as claudin-2 (CLDN2) at 1.25 ng/mL and 2.5 ng/mL and reached statistical significance at 5 ng/mL (1.59 ± 0.19 folds, P<0.005) as shown in **Figure 3B**.

### rIL-6 augments oxidative stress in intestinal cells while suppressing their apoptosis

**Figure 4:**
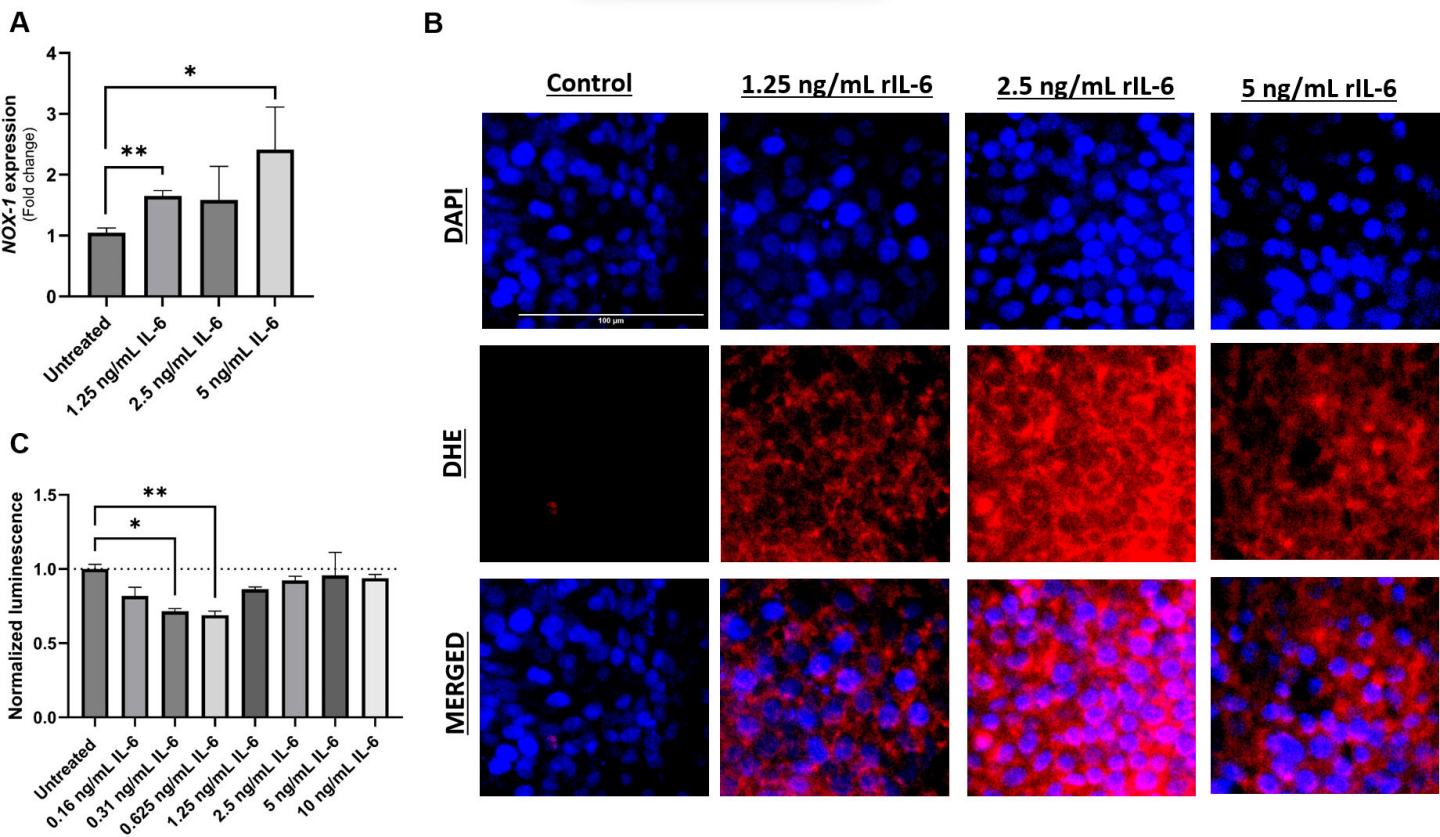
Effect of rIL-6 treatment on intestinal cell apoptosis and oxidative stress. HT-29 cells were treated with different concentrations of rIL-6 (1.25, 2.5 or 5 ng/mL) for 24 hours followed by qRT-PCR for NOX-1 **(A)** or DHE fluorescence staining for oxidative stress **(B)**. **(C)** Caco-2 cells were treated with a range of concentrations of rIL-6 (0.16 – 10 ng/mL) for 24 hours followed by an annexin V-luminescence assay for apoptosis determination. *Indicates P-value < 0.05 and **indicates P-value < 0.005.

NOX-1 is the catalytic subunit of the NADPH oxidase complex 1, a transmembrane protein abundant in colonic epithelium and responsible for the production of superoxide (O_2_^-^) which contributes to the cellular oxidative stress (24). Intestinal epithelial cells (Caco-2 and HT-29) were exposed to different concentrations of rIL-6 in culture for 24 hours. Analysis of NOX-1 gene expression in rIL-6-treated HT-29 revealed a concentration-dependent upregulation in the gene products following rIL-6 treatment (**Figure 4A**). This was evident at 1.25 ng/mL and 5 ng/mL, where rIL-6 treatment increased NOX-1 expression by 1.65 ± 0.09 folds (P-value < 0.005) and 2.42 ± 0.7 folds (P-value < 0.05), respectively. This was further confirmed in DHE staining which detects the presence of superoxide and hydrogen peroxide as shown in **Figure 4B**. Finally, we assessed the effect of rIL-6 treatment on cellular apoptosis as a consequence of oxidative stress. **Figure 4C** shows that rIL-6 treatment at 0.31 ng/mL and 0.625 ng/mL reduces the apoptosis in Caco-2 cells by 71% ± 2% (P-value < 0.05) and 69% ± 3% (P-value < 0.005), respectively.

### PAI-1 (SERPINE1) expression is upregulated in intestinal cell line following rIL-6 treatment

**Figure 5:**
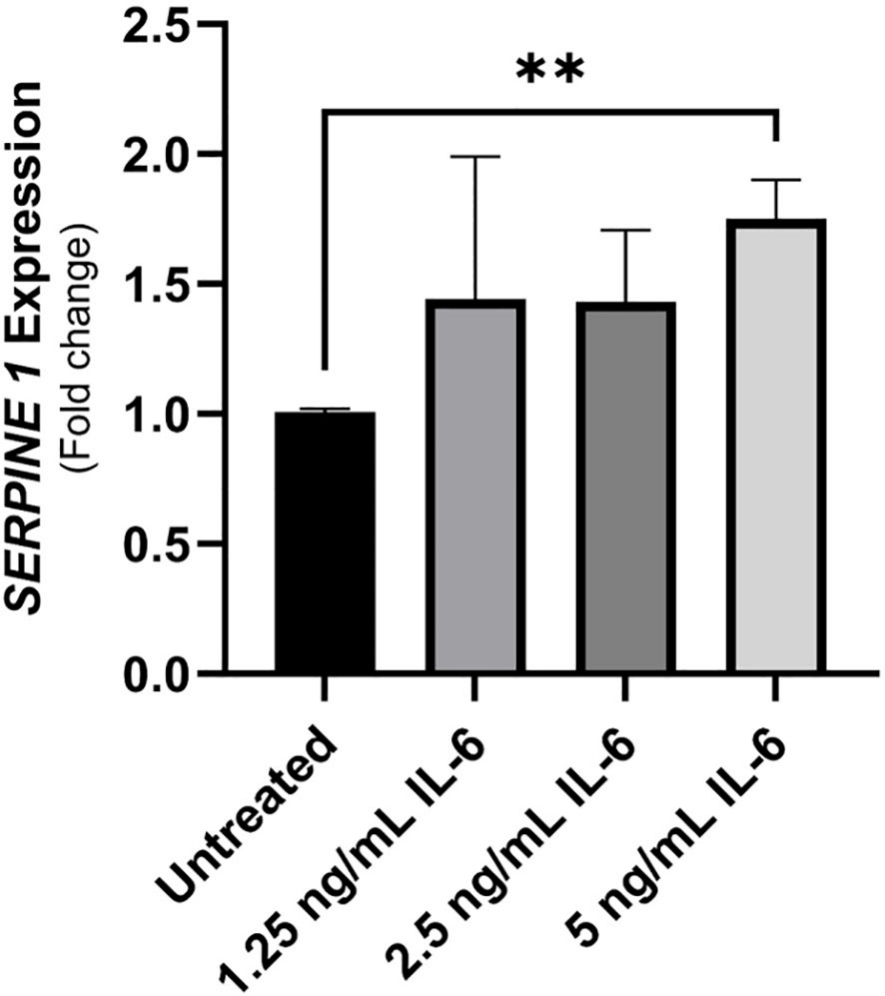
The implication of treating intestinal cells with rIL-6 on the expression of plasminogen-activator inhibitor (PAI-1)/SERPINE1. Caco-2 were treated with different concentrations of rIL-6 (1.25, 2.5 and 5 ng/mL) for 24 hours and then extracted RNA was used in a qRT-PCR to detect SERPINE1/PAI-1 expression compared to an untreated control. **Indicates P-value < 0.005.

The plasminogen activator inhibitor-1 (PAI-1 also known as SERPINE1) is a mucosal damage predictor that leads to enhanced inflammation and mucosal damage due to reduced activation of the anti-inflammatory cytokine TGFβ (25). Caco-2 were treated with different concentrations of rIL-6 (1.25, 2.5 and 5 ng/mL) for 24 hours and then extracted RNA was used in a qRT-PCR to detect SERPINE1/PAI-1 expression compared to an untreated control. **Figure 5** shows an upregulation in SERPINE1/PAI-1 expression in Caco-2 cells treated with 5 ng/mL of rIL-6 compared to untreated cells (1.75 ± 0.15 folds of the control), P-value < 0.005. The effect was dose dependent.

### Neutralizing IL-6R alters tight junctions and mucin production during MAP infection

**Figure 6:**
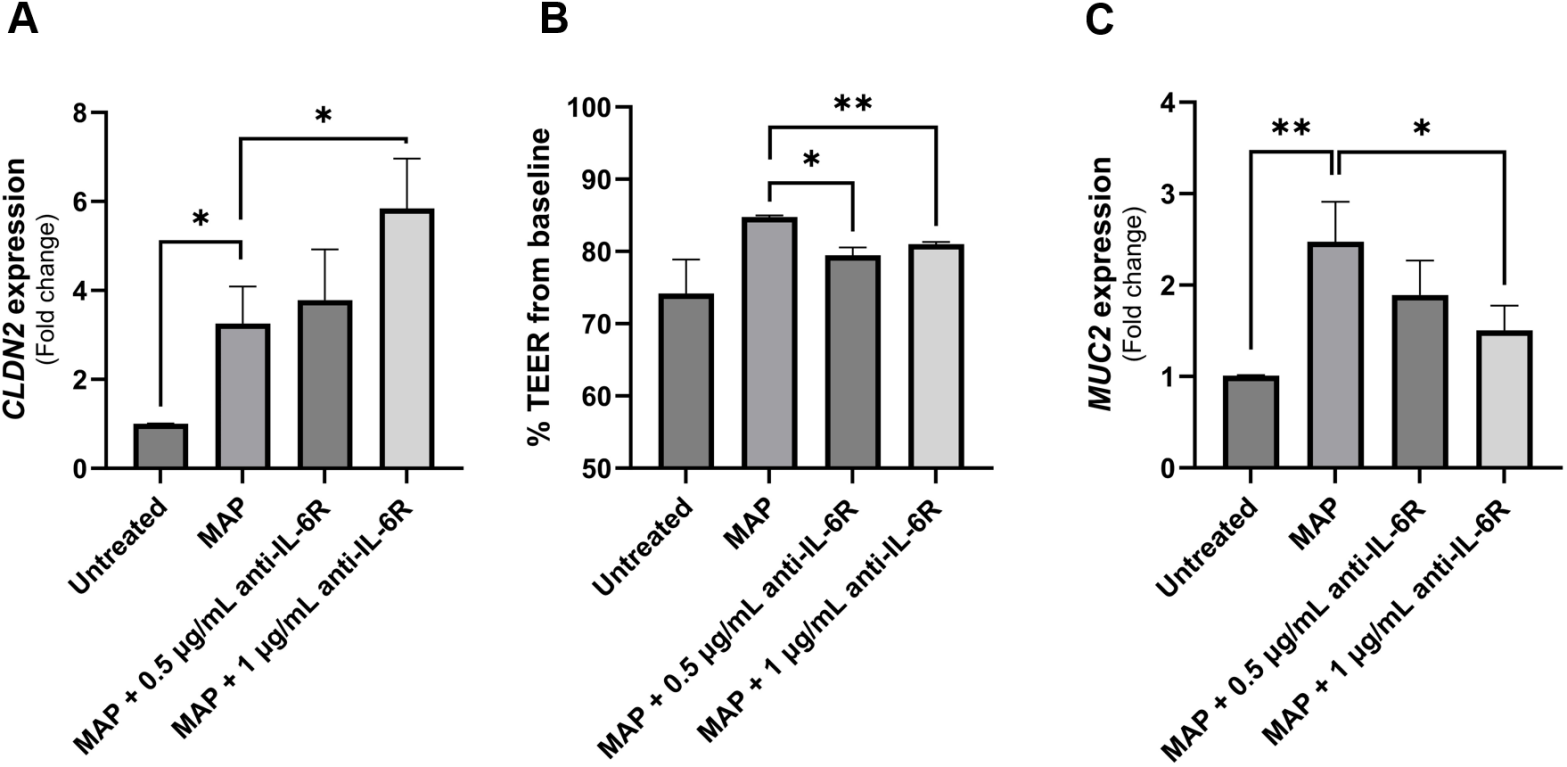
The impact of blocking IL-6 signaling during MAP infection on intestinal epithelial integrity markers. Caco-2 and HT-29 cells were treated with MAP supernatant alone or while double-blocked with anti-IL-6R antibody at different concentrations (0.5 and 1 µg/mL) for 1 hour. Caco-2 were used to analyze CLDN2 gene expression via qRT-PCR **(A)** and to measure TEER across the epithelial monolayer **(B)**. TEER is presented as percentage from baseline TEER measurements. **(C)** Treated HT-29 cells were used to analyze MUC2 gene expression using qRT-PCR. All experiments were performed after 24 hours of incubation with the respective supernatant. *Indicates P-values < 0.05 and **indicates P-values < 0.005.

To investigate the effect of blocking IL-6 signaling on the integrity and function of intestinal epithelial cells during MAP infection, we used an anti-IL-6R antibody-analogous to tocilizumab used to treat CD patients in a clinical trial (19). Intestinal cells (Caco-2 and HT-29 cells) were treated with supernatant of MAP-infected THP-1 macrophages that was double-blocked with anti-IL-6R. **Figure 6A** shows that MAP infection increases the expression of claudin-2 in Caco-2 monolayers by 3.26 ± 0.84 folds compared to uninfected cells (P-value < 0.05). Neutralizing IL-6R in supernatant of MAP-infected macrophages has further enhanced claudin-2 upregulation in receptor-blocked Caco-2 compared to unblocked group (5.84 ± 1.12 folds vs 3.26 ± 0.84 folds, respectively). To further explore the effects on membrane permeability and the integrity of tight junctions, we measured the transepithelial electrical resistance (TEER) in the same cells. Blocking IL-6R during MAP infection reduced the TEER of Caco-2 monolayers indicating an increase in the permeability of the intestinal cell layer to ionic solutes (**Figure 6B**). As seen in **Figure 6C**, MAP infection upregulates the expression of MUC2 in HT-29 intestinal cells (2.47 ± 0.44 folds, P-value < 0.005) while blocking IL-6R during MAP infection reduces MUC2 expression by 1.51 ± 0.27 folds compared to MAP-infected cells (P-value < 0.05).

### Neutralizing IL-6R increased oxidative stress and modulated apoptosis in intestinal cell lines during MAP infection

**Figure 7:**
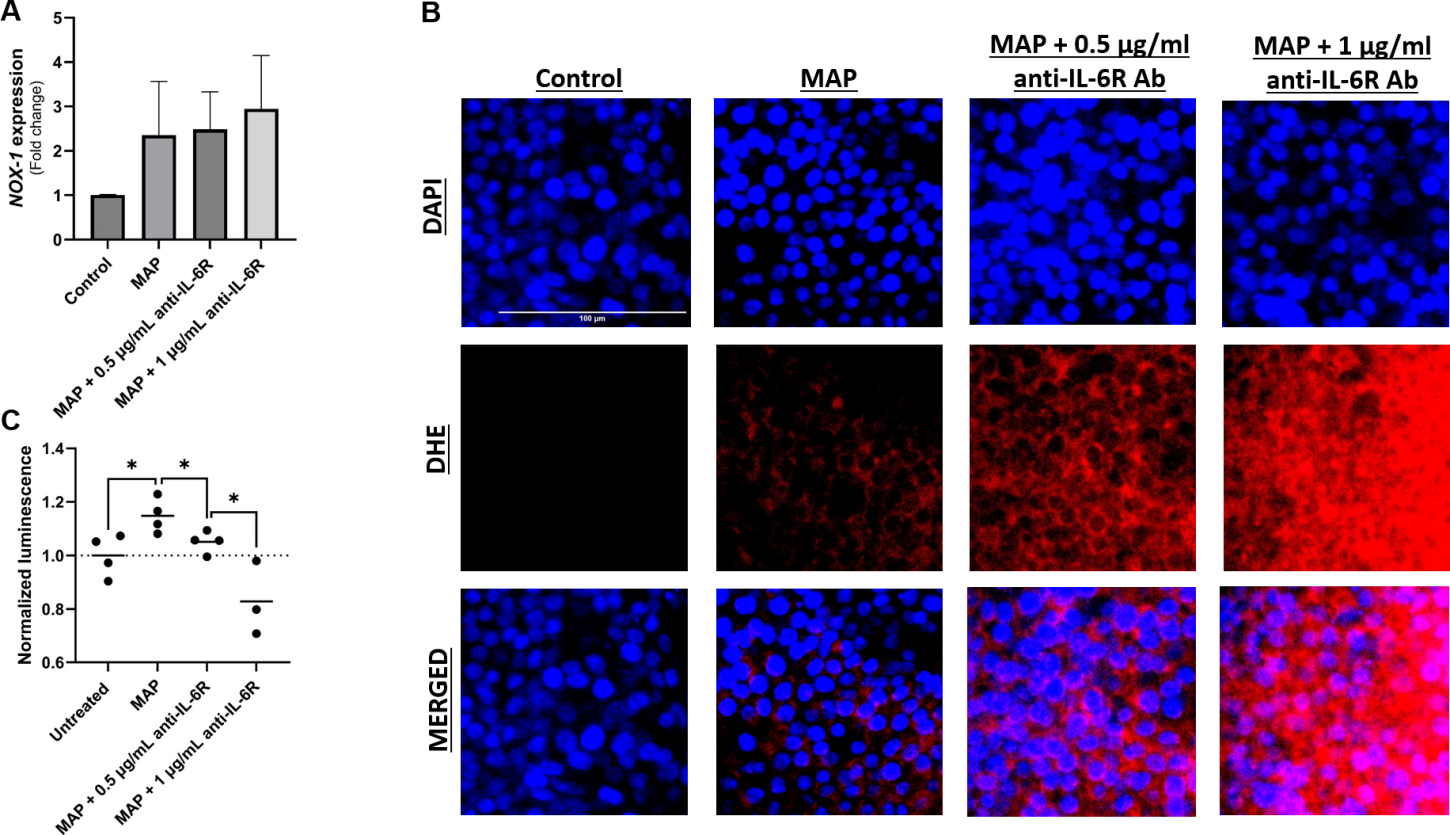
Effect of blocking IL-6 signaling during MAP infection on intestinal cell apoptosis and oxidative stress. Caco-2 and HT-29 cells were treated with MAP supernatant alone or while double-blocked with anti-IL-6R antibody at different concentrations (0.5 and 1 µg/mL) for 1 hour. 24 hours later, HT-29 cells were used to analyze NOX-1 gene expression via qRT-PCR **(A)** and to visualize cellular oxidative stress **(B)** stained with DHE (red) while Caco-2 cells were used in annexin V-based luminescence assay to quantify apoptosis **(C)**. *Indicates P-values < 0.05.

To further examine the role of IL-6 signaling during MAP infection on intestinal epithelial integrity, we assessed oxidative stress and the apoptotic capacity of challenged intestinal cells. As expected, MAP infection upregulated NOX-1 expression, however, blocking IL-6R in these infected cells has increased NOX-1 expression (**Figure 7A**). This was confirmed by DHE staining which shows an increase in the oxidative damage following IL-6R neutralization with 0.5 µg/ml and 1 µg/ml of antibody compared to unblocked MAP-infected cells (**Figure 7B**). Additionally, we observed that MAP infection is increasing cell apoptosis by 1.15 ± 0.06 folds (P-value < 0.05) as seen in **Figure 7C**. Blocking IL-6R reduced apoptosis by 1.05 ± 0.04 folds at 0.5 µg/mL (P-value < 0.05) and 0.83 ± 0.14 folds at 1 µg/mL (P-value < 0.05) compared to unblocked MAP-infected cells (**Figure 7C**).

### Neutralizing IL-6R increased SERPINE1/PAI-1 in intestinal cell lines during MAP infection

**Figure 8:**
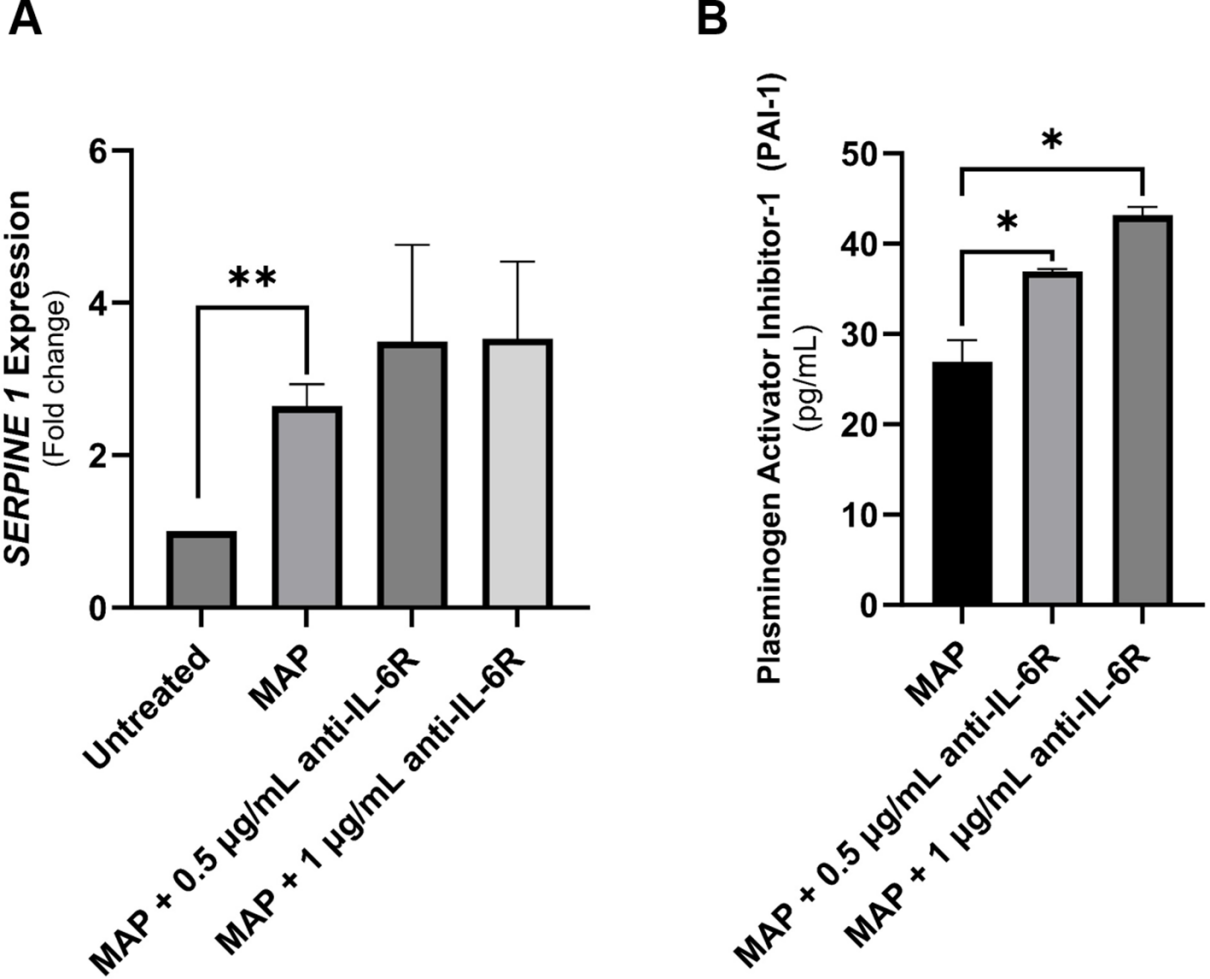
The production of SERPINE1/PAI-1 by intestinal epithelial cells following neutralizing IL-6R during MAP infection. **(A, B)** Caco-2 cells were treated with MAP supernatant alone or while double-blocked with anti-IL-6R antibody at different concentrations (0.5 and 1 µg/mL) for 1 hour. Following a 24-hour incubation, RNA and supernatants were collected to assay SERPINE1/PAI-1 mRNA expression (qRT-PCR) and protein products (ELISA), respectively. *Indicates P-values < 0.05 and **indicates P-values < 0.005.

To further determine if blocking IL-6 signaling will worsen the IBD damage markers especially in cases associated with MAP infection, we analyzed the expression of SERPINE1/PAI-1 by qRT-PCR and ELISA in cell lysates and supernatants of cultured Caco-2 cells exposed to MAP infection. As shown in **Figure 8**, SERPINE1/PAI-1 expression was upregulated during MAP infection 2.45 ± 0.29 folds (P-value < 0.05). This was not opposed by the blockade of IL-6R (**Figure 8A**). At the protein level, PAI-1 released from Caco-2 cells is evidently upregulated after IL-6R blockade with 0.5 µg/mL (36.95 ± 0.23 pg/mL, P-value < 0.05) and 1 µg/mL (43.13 ± 0.95 pg/mL, P-value < 0.05) during MAP infection, compared to uninterrupted MAP infection (26.94 ± 2.38 pg/mL) as seen in **Figure 8B**.

### Differential effects of rTNFα and rIL-6 versus MAP infection on intestinal epithelial integrity markers

**Figure 9:**
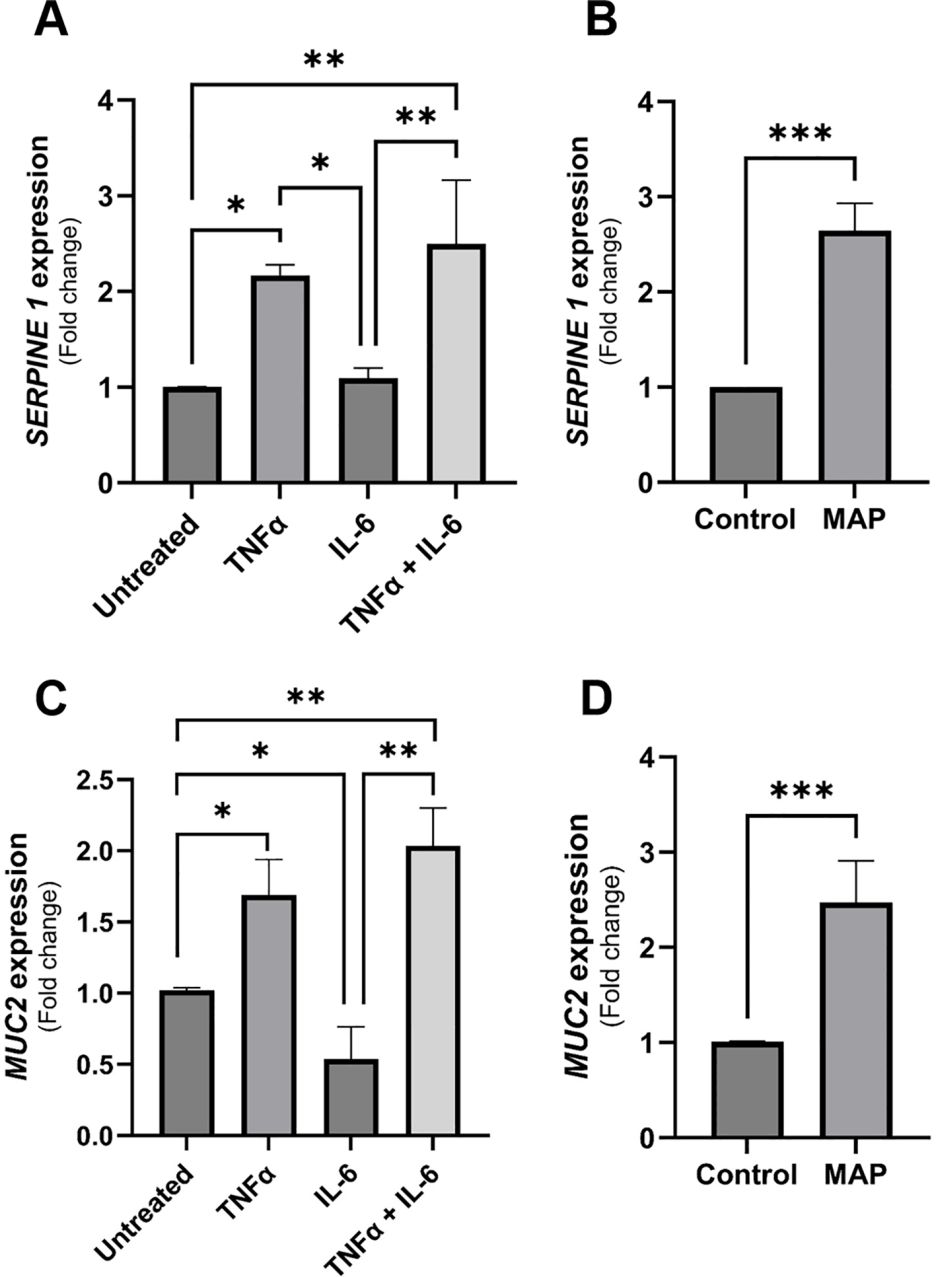
The comparative effects of rTNFα treatment on epithelial cells integrity markers compared to rIL-6. **(A)** Caco-2 cells were treated with 5 ng/mL of rTNFα or/and 5 ng/mL of rIL-6 for 24 hours followed by qRT-PCR for SERPINE1/PAI-1 gene expression. **(B)** Caco-2 cells were incubated with MAP-infected THP-1 supernatant or left untreated for 24 hours followed by qRT-PCR for SERPINE1/PAI-1 gene expression. **(C)** HT-29 cells were treated with 5 ng/mL of rTNFα or/and 5 ng/mL of rIL-6 for 24 hours followed by qRT-PCR for MUC2 gene expression. **(D)** HT-29 cells were incubated with MAP-infected THP-1 supernatant or left untreated for 24 hours followed by qRT-PCR for MUC2 gene expression. *Indicates P-values < 0.05, **indicates P-values < 0.005 and ***indicates P-values < 0.001.

Due to the observed differences between rIL-6 treatment and MAP infection on damage and functional markers of intestinal cells, we treated intestinal cells (Caco-2 and HT-29 cells) with equal concentrations of rIL-6 or/and rTNFα (5 ng/mL) and assessed marker expression using qRT-PCR after 24 hours of treatment. rTNFα upregulates SERPINE1/PAI-1 2.17 ± 0.11 folds (P-value < 0.05) compared to control, which is higher than rIL-6 treatment alone (**Figure 9A**). The combination of the two cytokines has an up-regulatory effect (2.5 ± 0.66 folds, P-value < 0.005) that almost matches that of MAP infection (2.65 ± 0.29 folds, P-value < 0.001) (**Figures 9A, B**). Regarding MUC2 in HT-29 cells, rTNFα treatment is augmenting the expression of MUC2 (1.69 ± 0.25 folds, P-value < 0.05) while rIL-6 is reducing it (0.54 ± 0.22 folds, P-value < 0.05) (**Figure 9C**). Lastly, the combined effect of both cytokines is enhancing MUC2 expression by 2.03 ± 0.27 folds (P-value < 0.005) which is nearing the effect of MAP infection (2.47 ± 0.44 folds, P-value < 0.001) (**Figures 9C, D**).

## Discussion

With more than half of CD patients may be infected with MAP, current treatment plans based on anti-inflammatory drugs are not effective for treatment of large population of CD patients (8, 26). It has been estimated that 10-40% of IBD patients show no primary response to anti-TNFα and about 50% lose response over time (27). Moreover, our group has demonstrated that anti-TNFα based therapy enhances the viability of MAP in patient macrophages which ultimately may worsen the condition of the patient and complicates the symptoms (28, 29). Recently, anti-IL-6 monoclonal antibodies including tocilizumab (TCZ) have been considered in clinical trials. Despite an improvement in disease activity indices in patients treated with TCZ, clinical trials outcome reported serious side effects like gastrointestinal bleeding and intestinal perforations in a subset of patients which led to discontinuation of these trials (17, 19). Clearly, alternative treatment options of inflammatory diseases are urgently needed. Here, we sought to investigate the effect IL-6 has on intestinal epithelial cells and the consequences of neutralizing IL-6R on intestinal epithelial health and disease markers especially in cases associated with MAP-induced inflammation.

Our findings demonstrate a debilitating effect due to IL-6R blockade on intestinal epithelial health and integrity especially during MAP infection. Neutralizing IL-6 signaling using an anti-IL-6R antibody as seen in clinical trials using TCZ disrupts intestinal tight junctions by upregulating the leaky protein, claudin-2, and reducing TEER measurements. Such treatment also reverses the upregulation of mucous layer glycoprotein, MUC2, during infection. Moreover, this blockade results in an increased cellular oxidative stress evident by NOX-1 expression and DHE staining as well as an augmentation of SERPINE1/PAI-1 release which is a biomarker of active disease and poor therapeutic response (30). These findings demonstrate the need for active IL-6 signaling during MAP infection to preserve intestinal epithelial integrity and health during infection and would shift therapeutic strategies to mechanisms that finetune IL-6 levels instead of eliminating it.

In this study, we have observed an upregulation of IL-6 in the plasma of MAP-positive CD patients compared to MAP-negative CD patients and healthy controls (**Figure 1**) and in the supernatants of MAP-infected THP-1 macrophages (**Figure 2A**). IL-6R, responsible for eliciting IL-6 signaling in target cells, is expressed on the surface of few cell types (e.g. hematopoietic cells, hepatocytes and some epithelial cells) and is also released from these cells in a soluble form, soluble IL-6R (sIL-6R), by the enzymatic cleavage of membrane receptors or the alternative splicing of IL-6R mRNAs (27, 31, 32). Analysis of sIL-6R levels in the supernatants of MAP-infected THP-1 macrophages, showed a significant increase during MAP infection, which is consistent with upregulated IL-6 signaling during MAP infection since sIL-6R activates the trans-signaling IL-6 pathway (16). Based on these findings, we have also assessed the direct effects of rIL-6 on intestinal epithelial integrity. Interestingly, treatment of cultured intestinal epithelial cells with different concentrations of rIL-6 proves to be damaging in our *in vitro* model. This is illustrated by the reduction in MUC2 expression and the surge in damage markers CLDN2, PAI-1 and NOX-1. However, the extent of intestinal epithelial damage and modulation to these markers varied between the level of rIL-6 treatment dosage and height of MAP infection. In **Figure 9**, we show how rTNFα alone or in combination with rIL-6 inflict similar effects to that of MAP infection which is reasonable given that both cytokines in addition to other inflammatory cytokines like IL-1β are upregulated during MAP infection. The synergetic effect of these cytokines may lead to cytokine-like storm in some CD patients infected with MAP infection.

Tight junction proteins, especially claudins, are key players in intestinal epithelial barrier function and integrity (33). Claudin-2 mediates the paracellular flux of water in leaky epithelia and contributes to the diarrheal consequences of intestinal inflammation (34, 35). Here, we show that neutralizing IL-6R during MAP infection further upregulates claudin-2 expression by intestinal cells which correlates to a reduced TEER measurement. We also demonstrate for the first time the direct damaging effect of MAP infection on claudin-2 expression by intestinal cells indicating the role of MAP infection in compromising the barrier function of intestinal epithelia. This is in alignment with previous work on other intestinal microbes like adherent-invasive *E. coli* and *Salmonella* as well as microbial products like cholera toxin and Staphylococcus enterotoxin B, and their effect on the upregulation of claudin-2 expression by intestinal cells in culture or mice models which correlates with reduced intestinal permeability (36–38). Liu et al. further elaborated another mechanism by which claudin-2 contributes to barrier dysfunction, where claudin-2 binds to luminal antigens and possibly facilitates their transport across the epithelium and can thus contribute to allergen-induced intestinal hypersensitivity (36). This and other findings from Zhang et al. provide evidence to a microbial-induced upregulation of gut permeability through the upregulation of claudin-2 which further facilitates the transport of microbes across the epithelial barrier and perpetuates inflammation (38). MAP could as well be using a similar mechanism to invade the epithelium and blocking IL-6R will only make it easier for MAP and other luminal microbes. This highlights claudin-2 as a potential therapeutic target for the treatment and alleviation of inflammation for MAP-infected CD patients.

MUC2 is the predominant secretory mucin of the intestine which forms a protective physical gel-like layer on intestinal epithelia impeding the invading pathogens (39, 40). Here, we show that MUC2 is upregulated by MAP infection, a normal response to an intestinal pathogen as seen with *E. coli* infection (40). This protective response is, however, counteracted by the blockade of IL-6R, where we have shown a reversal in MUC2 expression levels almost to that expected in control. Despite an observed inhibitory role of rIL-6 on MUC2 expression (**Figure 3A**), MAP infection resulted in a contrasting outcome. That could be explained by the effect of other inflammatory cytokines upregulated by MAP infection like TNFα, which we have demonstrated and compared its effect to that of IL-6. Indeed, rTNFα led to upregulation of MUC2 expression even when combined with rIL-6 (**Figure 9C**). Our findings are in agreement with Li et al. where they demonstrated a downregulation of MUC2 production by colon cancer cells due to rIL-6 or macrophage-derived IL-6 treatment in a STAT3-dependent manner (41). Interestingly, some research suggests that a related subspecies to MAP, *Mycobacterium avium hominissuis*, can avoid the mucin barrier and interact with intestinal epithelia in the presence or absence of MUC2 (42). This was attributed to cell wall structures and surface lipids. We are intrigued by this, and it would be compelling to investigate this observation during MAP infection. We anticipate that it would render the alterations to MUC2 production and consequence effects on the intestinal integrity and function during MAP infection irrelevant.

One of the constraints of our findings is the lack of an *in vivo* model for MAP infection to simulate real scenarios and assess consequences of epithelial damage in the intestine following IL-6R neutralization. Clearly, any *in vitro* model lacks the contributions of other immune cells, stromal elements, additional cytokines and regulators, and the role of intestinal microbiome. There is no doubt that these factors may influence IL-6 signaling and response to IL-6R neutralization. However, this is the first study that sheds new lights and provides valuable insights into the role of IL-6 in inflammatory response and intestinal epithelial damage and recovery during MAP infection in CD-like macrophages and epithelial cells.

In conclusion, we demonstrate in this study the damaging effects of neutralizing IL-6R on intestinal epithelial homeostasis during MAP infection which may explain the lack of effective therapeutic response in many CD patients receiving anti-IL-6 based therapy. In fact, our study demonstrates for the first time how neutralizing IL-6R exacerbates the inflammation and delays the recovery of the damaged intestine *in vitro* which may explain the side effects reported in patients receiving anti-IL-6 based therapy. We plan to focus on a therapeutic option that aims at modulating the IL-6 level during MAP infection to a physiological level that promotes IL-6 to play its dual homeostatic role as it was always intended.

## Data availability statement

The original contributions presented in the study are included in the article/supplementary material. Further inquiries can be directed to the corresponding author.

## Ethics statement

The studies involving humans were approved by University of Central Florida Internal Review Board committee. The studies were conducted in accordance with the local legislation and institutional requirements. Written informed consent for participation was not required from the participants or the participants’ legal guardians/next of kin because All plasma samples used in this study were approved as exempt.

## Author contributions

AA: Writing – original draft, Methodology, Investigation, Formal analysis, Data curation, Conceptualization. SN: Writing – review & editing, Supervision, Methodology, Funding acquisition, Conceptualization.
